# Carriage frequency, phenotypic, and genotypic characteristics of methicillin-resistant *Staphylococcus aureus* isolated from dental health-care personnel, patients, and environment

**DOI:** 10.1038/s41598-017-07713-8

**Published:** 2017-08-07

**Authors:** Ahmed S. Khairalla, Reham Wasfi, Hossam M. Ashour

**Affiliations:** 1 Department of Microbiology & Immunology, Faculty of Pharmacy, Beni-Suef University, Beni-Suef, Egypt; 2 Department of Microbiology & Immunology, Faculty of Pharmacy, October University for Modern Sciences and Arts (MSA), Giza, Egypt; 3 Department of Biological Sciences, College of Arts and Sciences, University of South Florida St. Petersburg, St. Petersburg, Florida USA; 4 Department of Microbiology and Immunology, Faculty of Pharmacy, Cairo University, Cairo, Egypt

## Abstract

There is limited data on methicillin-resistant *Staphylococcus aureus *(MRSA) carriage in dental clinics. 1300 specimens from patients, health personnel, and environmental surfaces of a dental clinic in Egypt were tested for MRSA. Antibiotic susceptibility, biofilm formation, Staphylococcal protein A (*spa*) typing, SCC*mec* typing, and PCR-based assays were used to detect *mecA*, *mecC*, *vanA*, Panton-Valentine Leukocidin toxin (*PVL*), and toxic shock syndrome toxin-1 (*tst*) genes. Among 34 *mecA*-positive MRSA isolates, five (14.7%) were *PVL*-positive, seventeen (50%) were *tst*-positive, ten (29.4%) were *vanA*-positive, while none harboured *mecC*. MRSA hand carriage rates in patients, nurses, and dentists were 9.8%, 6.6%, and 5%. The respective nasal colonization rates were 11.1%, 6.7%, and 9.7%. 1.3% of the environmental isolates were MRSA-positive. Strong and moderate biofilm-forming isolates represented 23.5% and 29.4% of MRSA isolates. 24 MRSA isolates (70.6%) were multi-resistant and 18 (52.9%) harboured SCC*mec* IV. Among eight *spa* types, t223 (26.5%), t267 (23.5%), and t14339 (23.5%) were predominant. We noted an alarming genetic relatedness between 7 (20.6%) MRSA isolates and the epidemic EMRSA-15 clone, as well as a combined occurrence of *tst* and *PVL* in 3 (8.8%) isolates. Results suggest high MRSA pathogenicity in dental wards highlighting the need for more efficient surveillance/infection control strategies.

## Introduction

*Staphylococcus aureus* is an infectious human pathogen that can survive on inanimate environmental surfaces^1^. It can colonize skin, mucous membranes, and the anterior nares in about 30% of healthy individuals^2, 3^. Methicillin-resistant *Staphylococcus aureus* (MRSA) is associated with substantial morbidity and mortality in many regions of the world^2^. MRSA strains that can spread rapidly among patients are known as epidemic MRSA (EMRSA) strains^4^. At least 17 different EMRSA clones have been identified^5^. One of these clones, EMRSA-15, is of global health concern, because it is highly transmissible, with capability of spreading between different continents, which explains its dissemination from the UK (where it was first reported) to several other parts of the world^6^.

MRSA infections, especially its biofilm-forming variants, are often difficult to treat for a variety of reasons. Firstly, these infections are usually attributed to multiple virulence determinants, including the *lukF/S-PV* genes encoding the Panton-Valentine leukocidin (PVL) toxin and the *tst* gene encoding the toxic shock syndrome toxin-1 (TSST-1)^3^. Secondly, infections with biofilm-forming strains of MRSA are usually persistent and respond poorly to conventional antibiotic therapy^7^. Thirdly, MRSA strains possess high levels of resistance to multiple antibiotics as a result of both intrinsic and acquired mechanisms^8^, such as the *mecA*- or *mecC*-mediated methicillin resistance^9, 10^, and *vanA*-mediated vancomycin resistance^11^. It has to be highlighted that while *mecA* gene is located on a mobile staphylococcal cassette chromosome (SCC) element known as SCC*mec*, twelve different types of SCC*mec* (I to XII) have been defined to date, five of which (I to V) are globally distributed^12–16^.

MRSA can be health-care-associated MRSA (HA-MRSA) or community-associated MRSA (CA-MRSA)^17^. HA-MRSA infections are more common in individuals with predisposing risk factors, such as hospitalization or invasive medical procedure^18^. Many CA-MRSA infections still arise in individuals not exposed to these risk factors^18^. CA-MRSA strains tend to be susceptible to many non-β-lactam antibiotics, whereas HA-MRSA strains are normally resistant to many antibiotic classes^19^. Despite efforts, CA-MRSA infections are on the rise worldwide^20^. In general, CA-MRSA strains are considered to be more virulent, transmissible, and persistent than their HA-MRSA counterparts^21, 22^. On the genetic level, there are remarkable differences between the two categories. HA-MRSA strains usually carry SCC*mec* types I, II, or III, whereas the SCC*mec* types IV or V together with the *PVL* gene are strongly associated with CA-MRSA strains^22^. Various molecular typing techniques have been developed for effective epidemiological surveillance and control of MRSA, the most common of which are SCC*mec* typing, multilocus sequence typing (MLST), *Staphylococcus* protein A (*spa*) typing, pulsed-field gel electrophoresis (PFGE) typing, and PVL typing. In this regard, studies have already shown the cost-effectiveness and the efficacy of *spa*, SCC*mec*, and PVL techniques compared to PFGE and MLST^23, 24^.

MRSA can be transmitted through a variety of ways in dental settings. These can include one or more of the following: (1) direct contact with blood or saliva (2) indirect contact with contaminated instruments or environmental surfaces; and (3) exposure to microbial aerosols released from the oral cavity^25–27^. Therefore, it is likely that dental clinic surfaces and dental health-care personnel (DHCPs) contribute to MRSA transmission to patients or other DHCPs^28, 29^.

Compared to the number of studies on MRSA isolates from hospitals^30–33^, less attention has been paid to MRSA isolated from dental care settings. More specifically, data related to the genetic diversity and virulence gene determinants of clones in dental clinics in the region, including Egypt, is scarce. Similarly, little is known about the carriage frequency, the biofilm-forming capacity, and the antimicrobial resistance profiles of MRSA isolated from these settings. Therefore, with a focus on MRSA isolates from dental care settings in Egypt, the objectives of the current study were to: (i) determine the prevalence of these isolates in different dental wards; (ii) assess their carriage rates in patients, nurses, dentists, and environmental surfaces; (iii) determine their genetic lineages using SCC*mec* and *spa* genotyping techniques; (iv) characterize their antimicrobial resistance profiles by disk diffusion or agar dilution techniques; (v) determine the presence or absence of five genes (*mecA*, *mecC*, *vanA*, *tst*, and *PVL*) implicated in antimicrobial resistance or virulence; and (vi) investigate the biofilm-forming abilities of the isolates.

It is anticipated that a better understanding of virulence gene profiling and molecular characterization of the clones circulating in both community and hospital settings will help us to develop more effective management plans and control strategies for MRSA infections.

## Results

### Prevalence of MRSA and other staphylococci

In this study, a total of 1300 swab specimens were collected from six different wards within a dental clinic in Egypt, including: 1030 (79.2%) specimens from environmental surfaces and 270 (20.8%) specimens from hands (*n* = 182) and anterior nares (*n* = 88) of both patients and DHCPs. These 1030 specimens from environmental surfaces fall into two categories, those from clinical-contact surfaces (*n* = 602) and those from housekeeping surfaces (*n* = 428) (Table 1).

**Table 1:** Distribution of the recovered isolates by site of specimen.

Specimen site	No. (%) of specimens or isolates			
	Specimens collected *n* = 1300	Recovered isolates *n* = 863	Isolates positive for *S. aureu n* = 112*s*	Isolates positive for MRSA *n* = 34
**Personnel**				
Hand (patients)	61 (4.69)	44 (5.10)	17 (15.18)	6 (17.65)
Nares (patients)	27 (2.08)	21 (2.43)	12 (10.71)	3 (8.82)
Hand (nurses)	61 (4.69)	49 (5.68)	12 (10.71)	4 (11.76)
Nares (nurses)	30 (2.31)	26 (3.01)	11 (9.82)	2 (5.88)
Hand (dentists)	60 (4.62)	54 (6.26)	7 (6.25)	3 (8.82)
Nares (dentists)	31 (2.38)	32 (3.71)	10 (8.93)	3 (8.82)
Total personnel specimens	270 (20.77)	226 (26.19)	69 (61.60)	21 (61.76)
**Environmental surfaces**				
Clinical-contact surfaces				
Dental light arm	66 (5.08)	10 (1.16)	5 (4.46)	1 (2.94)
Dentist’s chair	128 (9.85)	91 (10.54)	9 (8.04)	3 (8.82)
Dentist’s drill	112 (8.62)	56 (6.49)	8 (7.14)	2 (5.88)
Dentist’s tool rack	97 (7.46)	68 (7.88)	2 (1.79)	—
Patient’s sink faucet	116 (8.92)	98 (11.36)	7 (6.25)	3 (8.82)
X-ray switch	83 (6.38)	60 (6.95)	1 (0.89)	—
*Housekeeping surfaces*				
Dentist/nurse hand washing sink	68 (5.23)	14 (1.62)	1 (0.89)	—
Disinfectant containers	46 (3.54)	31 (3.59)	2 (1.79)	—
Door knobs	93 (7.15)	72 (8.34)	3 (2.68)	3 (8.82)
Floors	75 (5.77)	44 (5.10)	2 (1.79)	1 (2.94)
Light switches	94 (7.23)	54 (6.26)	2 (1.79)	—
Nurses’ desks	52 (4.0)	39 (4.52)	1 (0.89)	—
Total environmental surface specimens	1030 (79.23)	637 (73.81)	43 (38.40)	13 (38.24)

A minus sign (−) denotes the absence of an attribute.

Based on biochemical properties, 112 isolates (8.6%) from the total specimens collected were *S. aureus*, and 290 isolates (22.3%) were coagulase-negative *Staphylococcus* (CoNS). From any specimen source, the CoNS isolates were more predominant than *S. aureus* counterparts. For example, the CoNS carriage rates in hand, nasal, and environmental specimens were 23.6% (43/182), 40.9% (36/88), and 20.5% (211/1030), respectively, while the respective rates for *S. aureus* were 19.8% (36/182), 37.5% (33/88), and 4.2% (43/1030).

The isolates recovered from housekeeping surfaces demonstrated a lower prevalence of *S. aureus* as compared to those recovered from clinical-contact surfaces; however, this difference was statistically non-significant [4.3% versus 8.4%; *P* = 0.053 by Fisher’s exact test]. For clinical contact surfaces, the dentists’ chairs had the highest prevalence of *S. aureus* (8%), followed by dentists’ drills (7.1%) and patients’ faucet sinks (6.2%). For housekeeping surfaces, door knobs had the highest prevalence of *S. aureus* (2.68%), followed by disinfectant containers, floors, and light switches, which had equal prevalence rates of 7.1% each.

Screening for methicillin-resistant isolates was performed by the disk diffusion method [using oxacillin (1 μg) and cefoxitin (30 µg] and was subsequently verified by PCR targeting the *mecA* and *mecC* genes. Among the identified *S. aureus* isolates, 21.4% (24/112) were resistant to both antibiotics (Table 2). On the other hand, four isolates (Table 2, IDs: 11, 15, 20 and 68) showed an oxacillin-sensitive/cefoxitin-resistant profile, while six isolates (Table 2, IDs: 2, 25, 35, 70, 71 and 93) were oxacillin-intermediate but cefoxitin-resistant. All 34 isolates were positive for MRSA as indicated by PCR, leading to a MRSA prevalence of 30.4% (34/112) among all recovered *S. aureus* isolates.

**Table 2:** Sources and characteristics of individual MRSA isolates (*n* = 34) in this study.

Isolate ID	Specimen Source	Dental clinic/ward	Phenotypic resistance profile^*a*^	Biofilm forming ability^*b*^	Genotypic characteristics^*c*^				
					*PVL*	*vanA*	*tst*	*spa* type^*d*^	SCC*mec* type
**Isolates recovered from** **personnel** (***n*** = **21**)									
1a	Hand (patient)	Dental surgery	FOX-OX	Moderate	−	ND	+	t14339	II
1b	Hand (patient)	Dental surgery	FOX-OX-VAN*	Moderate	−	−	−	t14339	V
2	Hand (patient)	Dental surgery	FOX-DO-CN-OX*	Weak	−	ND	−	t267	I
7	Hand (patient)	Prosthetic dentistry	FOX-DO-CN-LZD-OX-VAN	Strong	−	+	+	t267	IV
15	Hand (patient)	Operative dentistry	FOX-CD-E-CN-LZD-VAN	Moderate	−	+	−	t084	IV
71	Hand (patient)	Prosthodontics	FOX-E-OX*	Non	−	ND	−	t223	I
56	Nares (patient)	Dental surgery	FOX-C-DO-CN-OX	Moderate	−	ND	−	t267	IV
63	Nares (patient)	Prosthetic dentistry	FOX-CIP-CD-CN-LZD-OX-VAN*	Non	−	−	+	t3689	I
93	Nares (patient)	Prosthodontics	FOX-E-CN-LZD-OX*-VAN*	Strong	−	−	−	t084	IV
23	Hand (nurse)	Dental surgery	FOX-C*-DO-E-CN-OX-VAN*	Weak	−	−	+	t267	I
112	Hand (nurse)	Dental surgery	FOX-DO-E-CN-LZD-OX	Non	+	ND	+	t14339	IV
30	Hand (nurse)	Prosthetic dentistry	FOX-DO-CN-OX	Weak	−	ND	+	t223	I
44	Hand (nurse)	Operative dentistry	FOX-DO-CN-OX-VAN	Moderate	−	+	−	t1339	IV
58	Nares (nurse)	Dental surgery	FOX-CIP-CD-E-OX-VAN*	Non	−	−	−	t223	Non-typeable^*e*^
59	Nares (nurse)	Dental surgery	FOX-C-CIP-DO*-E-CN-OX-VAN*	Weak	−	−	+	t14339	I
111	Hand (dentist)	Dental surgery	FOX-CD*-DO-CN-OX	Non	+	ND	+	t8506	IV
109	Hand (dentist)	Periodontics	FOX-CD-DO-CN-OX-VAN	Non	+	+	−	t380	IV
95	Hand (dentist)	Endodontics	FOX-DO-CN-OX	Non	−	ND	−	t267	IV
65	Nares (dentist)	Prosthetic dentistry	FOX-CIP-CD-CN-LZD-OX-VAN*	Moderate	−	−	−	t3689	IV
68	Nares (dentist)	Operative dentistry	FOX-C*-CD-DO-E-CN-LZD-VAN	Moderate	−	+	−	t380	I
70	Nares (dentist)	Periodontics	FOX-C-CD-E-OX*	Non	−	ND	−	t223	I
**Isolates recovered from** **environmental surfaces** (***n*** = **13**)									
5	Dental light arm	Dental surgery	FOX-CIP-E-CN- OX	Strong	−	ND	+	t223	IV
11	Dentist’s chair	Dental surgery	FOX-CD-LZD-VAN	Strong	+	−	+	t14339	IV
13	Dentist’s chair	Periodontics	FOX-C*-DO-E-CN-LZD-OX-VAN	Strong	−	+	+	t223	IV
18	Dentist’s chair	Prosthetic dentistry	FOX-C*-CD-DO-OX-VAN	Strong	−	−	+	t223	I
35	Dentist’s drill	Periodontics	FOX-C-DO-CN-OX*-VAN	Moderate	−	+	+	t084	IV
42	Dentist’s drill	Operative dentistry	FOX-DO-E-CN-OX-VAN*	Strong	−	−	+	t267	IV
25	Patient’s sink faucet	Prosthetic dentistry	FOX-C*-E-OX*-VAN*	Weak	−	−	+	t14339	I
26	Patient’s sink faucet	Prosthetic dentistry	FOX-DO-CN-OX-VAN	Moderate	−	+	−	t223	I
50	Patient’s sink faucet	Periodontics	FOX-C*-E-OX-VAN*	Weak	−	−	+	t14339	I
20	Door knob	Periodontics	FOX-E-CN-VAN*	Moderate	−	−	+	t267	IV
33	Door knob	Operative dentistry	FOX-C*-DO-E-CN-OX-VAN	Strong	−	+	−	t14339	I
103	Door knob	Endodontics	FOX-C-DO-E-CN-OX	Non	+	ND	−	t223	IV
38	Floor	Operative dentistry	FOX-C-DO-E-CN-OX-VAN*	Weak	−	−	−	t267	IV

*PVL*: The gene encoding the Panton–Valentine leucocidin toxin; *vanA:* the gene encoding an enzyme that causes a structural change in the terminal amino acid of the pentapeptide chain of peptidoglycan, thus conferring vancomycin resistance; *tst*: the gene encoding the toxic shock syndrome toxin; *spa:* staphylococcal protein A; SCC*mec*: staphylococcal cassette chromosome *mec*; a plus sign (+) denotes the presence of a gene; a minus sign (−) denotes the absence of a gene; ND: not determined.

^*a*^FOX: cefoxitin, C: chloramphenicol, CIP: ciprofloxacin, CD: clindamycin, DO: doxycycline, E: erythromycin, CN: gentamicin, LZD: linezolid, OX: oxacillin, and VAN: vancomycin

*Denotes intermediate resistance to the antibiotic, according to CLSI guidelines and breakpoints^88^.

^*b*^The isolates were classified as biofilm non producers, weak, moderate, and strong biofilm producers based on previously published criteria^34^.

^*c*^All the MRSA isolates investigated in the current study were *mecA-*positive and *mecC-*negative.

^*d*^The *spa* types shown are based on the Ridom StaphType software.

^*e*^Non-typeable: An isolate that was positive only for the *mecA* gene, with no PCR product obtained, or not in agreement with the predicted band patterns of SCC*mec* types I-V by the multiplex PCR method used.

The prevalence rate of MRSA was 0.98% (2/205) in samples collected from the endodontic ward, 2.9% (6/205) in samples from the operative dentistry, 2.4% (6/255) in samples from the periodontics, 3.9% (7/180) in samples from the prosthetic dentistry, 1% (2/200) in samples from the prosthodontics, and 4.3% (11/255) in samples from the dental surgery ward (Tables 2 and 3). This prevalence difference was found to be statistically non-significant (χ^2^ = 8.394, df = 5, *P* = 0.136).

**Table 3:** Ward distribution of the specimens collected in this study.

Dental ward	No. (%) of specimens or isolates					
	**Personnel**		**Environmental surfaces**		**Total no. (Personnel + Environmental)**	
	Specimens collected *n* = 270	Recovered isolates *n* = 226	Specimens collected *n* = 1030	Recovered isolates *n* = 637	Specimens collected *n* = 1300	Recovered isolates *n* = 863
Endodontics	38 (14.02)	32 (14.16)	167 (16.21)	126 (19.78)	205 (15.77)	158 (18.31)
Operative dentistry	40 (14.81)	26 (11.51)	165 (16.02)	104 (16.33)	205 (15.77)	130 (15.06)
Periodontics	45 (16.7)	28 (12.39)	210 (20.39)	103 (16.17)	255 (19.62)	131 (15.18)
Prosthetic dentistry	49 (18.15)	48 (21.24)	131 (12.72)	90 (14.13)	180 (13.85)	138 (15.99)
Prosthodontics	48 (17.8)	46 (20.35)	152 (14.76)	118 (18.52)	200 (15.38)	164 (19.00)
Dental surgery	50 (18.52)	46 (20.35)	205 (19.90)	96 (15.07)	255 (19.61)	142 (16.46)
Total	270 (100)	226 (100)	1030 (100)	637 (100)	1300 (100)	863 (100)

As shown in Table 1, the highest hand carriage rate of MRSA was detected in patients (9.8%, 6/ 61), followed by nurses (6.6%, 4/61), and dentists (5%, 3/60); however, this difference was not statistically significant (χ^2^ = 2.006, df = 2, *P* = 0.3666). The highest MRSA nasal colonization rate was observed in patients (11.1%, 3/27), followed by dentists (9.7%, 3/31), and nurses (6.7%, 2/30) (Table 1). This difference was also non-significant (χ^2^ = 0.5883, df = 2, *P* = 0.7452).

The environmental surfaces in 5 (83.3%) out of the 6 wards under study were contaminated with MRSA (Table 2). Environmental surfaces within the prosthetic dentistry ward showed the highest prevalence (2.3%, 3/131) of MRSA, followed by those from periodontics (1.9%, 4/210), operative dentistry (1.8%, 3/165), dental surgery (0.98%, 2/205), endodontics (0.60%, 1/167), while those within the prosthodontic ward were MRSA-free. This difference turned to be statistically significant (χ^2^ = 6.42, df = 5, *P* = 0.2675). The highest prevalence of MRSA in the environmental surfaces was observed in door knobs (3.2%, 3/93) and dentists’ chairs (2.3%, 3/128), while the lowest prevalence was found in dental light arms (1.5%, 1/66) and floors (1.3%, 1/75).

### Characterization of the MRSA isolates

#### Genetic groups based on *spa* typing and SCC*mec* typing

The *spa* typing analysis revealed 8 distinct *spa* types within the tested MRSA isolates. The *spa* type attribution of each isolate is reported in Table 2. The frequencies, geographical spread, and repeat successions for each identified *spa* type are reported in Table 4. The *spa* type including the largest number of isolates was t223 (*n* = 9, 26.5% of all tested MRSA isolates). This was immediately followed by t14339 and t267 (each of which contained 8 isolates, 23.5%). The other *spa* types were less frequent, including t084 (*n* = 3), t3689 (*n* = 2), t380 (*n* = 2), t8506 (*n* = 1), and t1339 (*n* = 1).

**Table 4:** Frequencies, geographical spread, repeat successions, and predicted clonal complexes (CCs) of the *spa* types detected in this study.

Cluster group and *spa*-CCs^*a*^	Ridom *spa* type^*b*^	No. (%) of MRSA isolates				Geographical spread*	*spa* repeat succession	Predicted MLST CC^*d*^
		Total specimens *n* = 34	Personnel specimens *n* = 21	Environmental surface specimens	Relative global frequency*			
Cluster 1 *spa*-CC223	t223	9 (26.5%)	4 (19%)	5 (38.5%)	0.42%	Austria, Belgium, Chile, Czech Republic, Denmark, Detmold, France, Germany, Iceland, Ireland, Israel, Italy, Jordan, Minden, Netherlands, New Zealand, Norway, Romania, Spain, Sweden, Switzerland, Syria, United Arab Emirates, United Kingdom, the Gaza Strip (Palestine)^50^, Kuwait^61^	26-23-13-23-05-17-25-17-25-16-28	CC22^*f*^
	t14339	8 (23.5%)	4 (19%)	4 (30.8%)	0.00%	Ireland	26-23-13-23-36-17-25-17-25-16-28	CC22
	t3689	2 (5.9%)	2 (9.5%)	—	0.00%	Denmark	26-23-13-23-05-17-25-17-25-25–16-28	CC22
	t8506	1 (2.9%)	1 (4.8%)	—	0.00%	Saudi Arabia	26-23-13-16-05-17-25-17-25-16-28	CC22
Singletons	t267	8 (23.5%)	5 (23.8%)	3 (23%)	0.30%	Argentina, Austria, Belgium, Cyprus, Denmark, France, Gabon, Germany, Iceland, Iran, Israel, Italy, Jordan, Lebanon, Netherlands, New Zealand, Norway, Saudi Arabia, South Africa, Spain, Sweden, Taiwan, United Arab Emirates, United Kingdom, United States	07-23-12-21-17-34-34-34-33-34	CC80^*f*^
	t084	3 (8.9%)	2 (9.5%)	1 (7.7%)	1.76%	Argentina, Australia, Austria, Belgium, China, Denmark, Finland, France, Gabon, Germany, Greece, Iceland, Indonesia, Iran, Italy, Jordan, Lebanon, Netherlands, New Zealand, Nigeria, Norway, Poland, Romania, South Africa, Spain, Sweden, Switzerland, Taiwan, Uganda, United Arab Emirates, United Kingdom, United States, Germany	07-23-12-34-34-12-12-23-02-12-23	CC15^*e*^
	t1339	1 (2.9%)	1 (4.8%)	—	0.01%	Austria, Denmark, Germany, Iceland, Norway, Sweden, United Arab Emirates	07-12-21-17-13-13-34-13-34-33-34	CC80^*g*^
Excluded	t380	2 (5.9%)	2 (9.5%)	—	0.00%	Germany, Lebanon, Sweden, United Kingdom	26-34-33-34	NP

^*^Based on data from the Ridom StaphType database (http://spa.ridom.de/frequencies.shtml), last accessed on 15 November 2016, unless otherwise stated. Countries from the Arab world are double-underlined.

^a^ *spa-*CC: denotes *spa* clonal complex as determined by the Based Upon Repeat Pattern (BURP) algorithm in the Ridom StaphType software with a distance cost of ≤ 5; excluded: denotes entries excluded from BURP clustering because the *spa* repeat pattern comprised fewer than five repeats.

^b^Nomenclature according to Harmsen *et al*.^91^; the *spa* types shown are based on the Ridom StaphType software.

^c^A minus sign (−) indicates the absence of the *spa* type.

^*d*^MLST CC: denotes predicted clonal complexes based on multi-locus sequence typing.

^*e*^According to the Ridom SpaServer (http://spaserver.ridom.de).

^*f*^Based on data from^98^.

^*g*^Based on data from^99^.

NP: not predictable (not yet assigned to CC).

Two of the most frequent *spa* types in our study (t223 and t267) were disseminated in different wards, since they were recovered from five out of the six tested wards (Table 5). Conversely, the *spa* type 14339 was mainly predominant in the dental surgery ward (62.5%, 5/ 8).

**Table 5:** Characteristics and detailed distribution of the *spa*-CCs and *spa*-types detected in this study.

Cluster group and *spa*-CCs	Ridom *spa* type^*a*^	No. of MRSA isolates																								
		*Based on specimen source*												*Based on dental ward*						*Based on presence of antibiotic resistance or virulence genes* ^***b***^			*Based on biofilm forming ability* ^***c***^			
		**Personnel** (*n* = 21)						**Environmental surfaces** (*n* = 13)						*End-odon-tics* (*n = 2*)	*Oper-ative denti-stry* (*n = 6*)	*Periodontics* (*n = 6*)	*Prost-hetic dent-istry* (*n = 7*)	*Prostho-dontics* (*n = 2*)	*Dental surgery* (*n = 11*)	*PVL* (*n = 5*)	*vanA* (*n = 10*)	*tst* (*n = 17*)	Non (*n = 9*)	Weak (*n = 7*)	Mode-rate (*n = 10*)	Strong (*n = 8*)
		**Patients**		**Nurses**		**Dentists**		**Clinical-contact**				**Housekeeping**														
		*Hand* (*n = 6*)	*Nares* (*n = 3*)	*Hand* (*n = 4*)	*Nares* (*n = 2*)	*Hand* (*n = 3*)	*Nares* (*n = 3*)	*D light arm* (*n = 1*)	*D chair* (*n = 3*)	*D drill* (*n = 2*)	*P sink faucet* (*n = 3*)	*Door knobs* (*n = 3*)	*Floors* (*n = 1*)													
Cluster 1 *spa*-CC223	**t223** (*n* = 9)	1	0	1	1	—	1	1	2	—	1	1	—	1	—	2	3	1	2	1	2	4	4	1	1	3
	**t14339** (*n* = 8)	2	—	1	1	—	—	—	1	—	2	1	—	—	1	1	1	—	5	2	2	6	1	3	2	2
	**t3689** (*n* = 2)	—	1	—	—	—	1	—	—	—	—	—	—	—	—	—	2	—	—	—	—	1	1	—	1	—
	**t8506** (*n* = 1)	—	—	—	—	1	—	—	—	—	—	—	—	—	—	—	—	—	1	1	—	1	1	—	—	—
Singletons	**t267** (*n* = 8)	2	1	1	—	1	—	—	—	1	—	1	1	1	2	1	1	—	3	—	1	4	1	3	2	2
	**t084** (*n* = 3)	1	1	—	—	—	—	—	—	1	—	—	—	—	1	1	—	1	—	—	2	1	—	—	2	1
	**t1339** (*n* = 1)	—	—	1	—	—	—	—	—	—	—	—	—	—	1	—	—	—	—	—	1	—	—	—	1	—
Excluded	**t380** (*n* = 2)	—	—	—	—	1	1	—	—	—	—	—	—	—	1	1	—	—	—	1	2	—	1	—	1	—

*spa-*CC: denotes *spa* clonal complex as determined by the Based Upon Repeat Pattern (BURP) algorithm in the Ridom StaphType software with a distance cost of ≤ 5; excluded: denotes entries excluded from BURP clustering because the *spa* repeat pattern comprised fewer than five repeats; *D light arm*: Dental light arm; *D chair*: Dentist’s chair; *D drill*: Dentist’s drill; *P sink faucet*: Patient’s sink faucet; *PVL*: The gene encoding the Panton–Valentine leucocidin toxin; *vanA:* the gene encoding an enzyme that causes a structural change in the terminal amino acid of the pentapeptide chain of peptidoglycan, thus conferring vancomycin resistance; *tst*: the gene encoding the toxic shock syndrome toxin; *spa:* staphylococcal protein A; a minus sign (−) denotes the absence of an attribute.

^*a*^Nomenclature according to Harmsen *et al*.^91^; the *spa* types shown are based on the Ridom StaphType software.

^*b*^All the MRSA isolates investigated in the current study were *mecA-*positive and *mecC-*negative.

^*C*^The isolates were classified as biofilm non-producers, weak, moderate, and strong biofilm producers based on previously published criteria^34^.

Only four *spa* types (t223, t14339, t267, and t084) coexisted in both personnel and environmental surface specimens, with higher frequencies of t267 and t084 in personnel specimens, an equal distribution of t14339 in both specimen categories, and higher frequency of t223 in environmental surface specimens (Table 5). On the other hand, the other four *spa* types (t3689, t8506, t1339, and t380) were only detected among isolates from personnel (Table 5).

**Figure 1:**
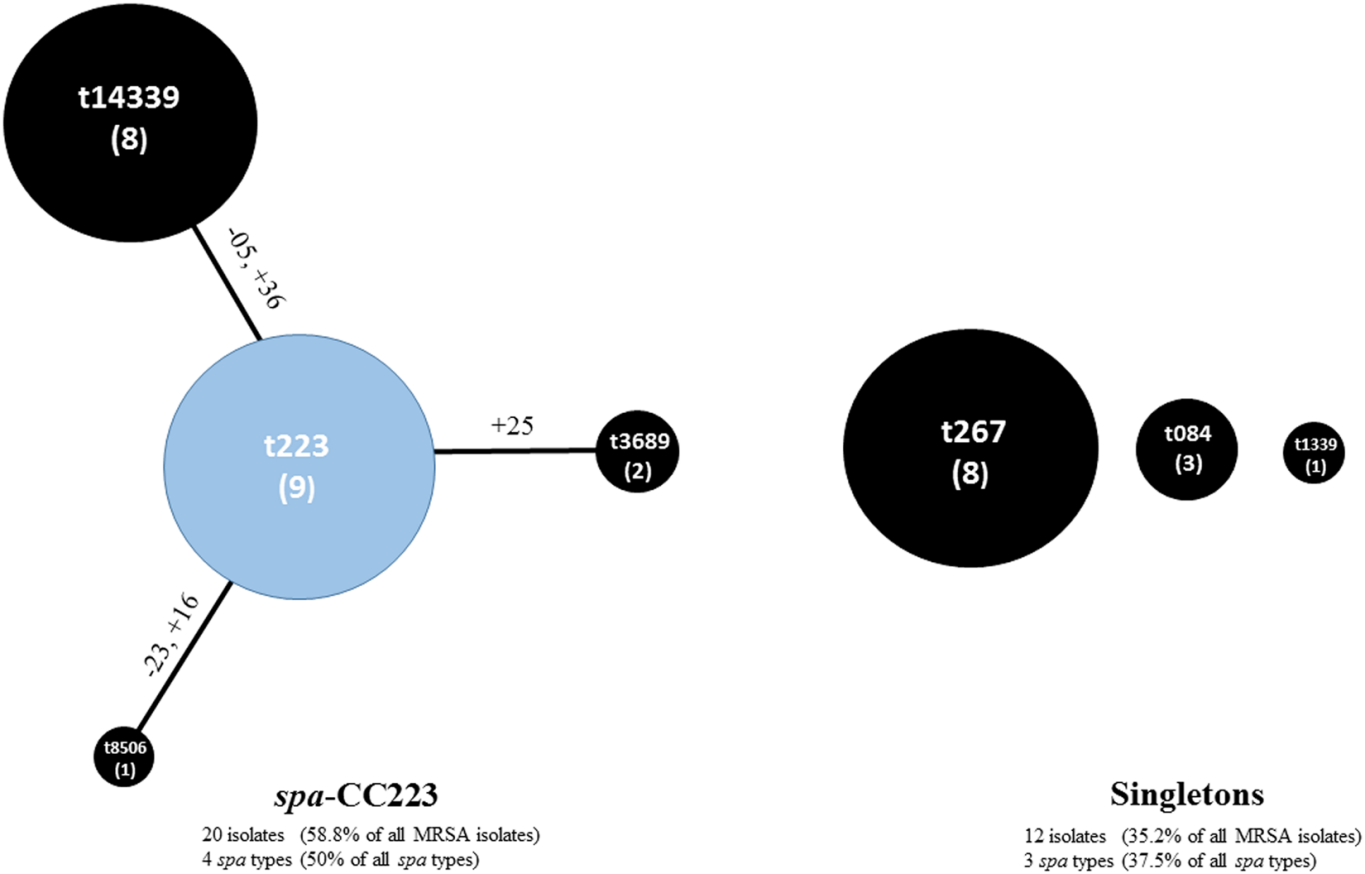
Population structure of the tested MRSA isolates (*n* = 34) based on BURP analysis. This analysis was performed using the Based Upon Repeat Pattern (BURP) algorithm of the Ridom StaphType software (Ridom GmbH, Würzburg, Germany) at a cost setting of ≤ 5 and excluding *spa*-types with 5 or fewer repeats. Each dot represents a different *spa* type, with the diameter of the dot being proportional to the quantity of the corresponding *spa* type. Clusters of linked *spa* types correspond to *spa* clonal complexes (*spa*-CCs). The predict founder of a cluster (which was used for defining the cluster) is shown in blue, while the others in black. Near the lines of connection, the mutations involved in the transition from a *spa* type to the next one are reported in detail. All DNA changes are meant to occur from the founder to the periphery. Legend: numbers along the lines refer to the repeat sequence involved in the mutation; +indicates the acquisition of a repeat sequence; - indicates the loss of a repeat sequence; within circles the numbers of the strains of each CC appear between brackets. In summary, the analysis identified a single clonal complex (*spa*-CC223) comprising *spa* types t223, t14339, t3689, and t8506; *n* = 20 isolates, and accounted for 58.8% of all tested MRSA isolates, as well as 3 singletons (t267, t084, and t1339; *n* = 12 isolates, 35.2%), while excluded 2 isolates (t380, 5.8%) from the clustering, as they consisted of four repeat units only.

The BURP algorithm (cost ≤ 5) assigned the isolates into a single clonal complex, *spa*-CC223 (*n* = 20 isolates, 58.8% of all tested MRSA isolates), as well as 3 singletons (*n* = 12 isolates, 35.2%), while excluding 2 isolates (5.8%) from the clustering (Table 4). The *spa* types in the *spa*-CC223 were: t223 (9 strains out of 20, 45%), t14339 (8, 40%), t3689 (2, 10%), t8506 (1, 5%) as presented in Fig. 1 and Table 4. Table 5 lists the characteristics and detailed distribution of different *spa*-CCs and *spa* types.

For SCC*mec* typing, the multiplex PCR assay identified 18 out of 34 MRSA isolates (52.9%) with SCC*mec* type IV, 13 (38.2%) with SCC*mec* type I, 1 isolate (2.9%) with SCC*mec* type II, and 1 isolate (2.94) with SCC*mec* type V (Table 6). The SCC*mec* type III was not found in any isolate. One isolate (2.9%) could not be typed (Table 6

**Table 6:** Characteristics and detailed distribution of the SCCmec types detected in this study.

SCC*mec* type	No. of MRSA isolates																								
	*Based on specimen source*												*Based on dental ward*						*Based on presence of antibiotic resistance or virulence genes* ^***a***^			*Based on biofilm orming ability* ^***b***^			
	**Personnel** (*n* = 21)						**Environmental surfaces** (*n* = 13)						*Endodontics* (*n = 2*)	*Operative dentistry* (*n = 6*)	*Periodontics* (*n = 6*)	*Prosthetic dentistry* (*n = 7*)	*Prosthodontics* (*n = 2*)	*Dental surgery* (*n = 11*)	*PVL* (*n = 5*)	*vanA* (*n = 10*)	*tst* (*n = 17*)	Non (*n = 9*)	Weak (*n = 7*)	Moderate (*n = 10*)	Strong(*n = 8*)
	**Patients**		**Nurses**		**Dentists**		**Clinical-contact**				**Housekeeping**														
	*Hand* (*n = 6*)	*Nares* (*n = 3*)	*Hand* (*n = 4*)	*Nares* (*n = 2*)	*Hand* (*n = 3*)	*Nares* (*n = 3*)	*D. light arm* (*n = 1*)	*D. chair* (*n = 3*)	*D. drill* (*n = 2*)	*P. sink faucet* (*n = 3*)	*Door knobs* (*n = 3*)	*Floors* (*n = 1*)													
I **(** ***n*** ** = 13)**	2	1	2	1	—	2	—	1	—	3	1	—	—	2	2	5	1	3	—	3	7	3	6	2	2
II **(** ***n*** ** = 1)**	1	—	—	—	—	—	—	—	—	—	—	—	—	—	—	—	—	1	—	—	1	—	—	1	—
III **(** ***n*** ** = 0)**	—	—	—	—	—	—	—	—	—	—	—	—	—	—	—	—	—	—	—	—	—	—	—	—	—
IV **(** ***n*** ** = 18)**	2	2	2	—	3	1	1	2	2	—	2	1	2	4	4	2	1	5	5	7	9	5	1	6	6
V **(** ***n*** ** = 1)**	1	—	—	—	—	—	—	—	—	—	—	—	—	—	—	—	—	1	—	—	—	—	—	1	—
Non-typeable **(** ***n*** ** = 1)**	—	—	—	1	—	—	—	—	—	—	—	—	—	—	—	—	—	1	—	—	—	—	—	—	—

SCC*mec*: staphylococcal cassette chromosome *mec*; Non-typeable: an isolate with no PCR product obtained, or not in agreement with the predicted band patterns of SCC*mec* types I-V by the multiplex PCR method used; *D light arm*: Dental light arm; *D chair*: Dentist’s chair; *D drill*: Dentist’s drill; *P sink faucet*: Patient’s sink faucet; *PVL*: The gene encoding the Panton–Valentine leucocidin toxin; *vanA:* the gene encoding an enzyme that causes a structural change in the terminal amino acid of the pentapeptide chain of peptidoglycan, thus conferring vancomycin resistance; *tst*: the gene encoding the toxic shock syndrome toxin; *spa:* staphylococcal protein A; a minus sign (−) denotes the absence of an attribute.

^*a*^All the MRSA isolates investigated in the current study were *mecA-*positive and *mecC-*negative.

^*b*^The isolates were classified as biofilm non-producers, weak, moderate, and strong biofilm producers based on previously published criteria^34^.

).

#### Antimicrobial resistance profile

Antimicrobial susceptibility testing of the MRSA isolates revealed thirty one resistance profiles, in which nineteen and thirteen of these profiles were observed among the MRSA isolates recovered from personnel and environmental surfaces, respectively (Table 2).

The rates of full resistance among the 34 MRSA isolates tested in the current study were as follows: 100% (*n* = 34) for cefoxitin, 17.6% (*n* = 6) for chloramphenicol, 14.7% (*n* = 5) for ciprofloxacin, 26.5% (*n* = 9) for clindamycin, 55.8% (*n* = 19) for doxycycline, 52.9% (*n* = 18) for erythromycin, 73.5% (*n* = 25) for gentamycin, 26.4% (*n* = 9) for linezolid, 67.6% (*n* = 23) for oxacillin, and 29.4% (*n* = 11) for vancomycin. Conversely, all tested isolates were susceptible to cefaclor, ceftriaxone, imipenem, and neomycin.

Some isolates showed intermediate resistance to the tested antimicrobials, with a rate of 20.6% (*n* = 7) for chloramphenicol, 2.9% (*n* = 1) for doxycycline, 17.6% (*n* = 6) for oxacillin, and 35.3% (*n* = 12) for vancomycin. The overall rates of resistance (defined as the rate of intermediate resistance plus the rate of full resistance) to the previously mentioned antimicrobial agents were as follows: 38.2% (*n* = 13) for chloramphenicol, 58.8% (*n* = 20) for doxycycline, 85.3% (*n* = 29) for oxacillin, and 67.6% (*n* = 23) for vancomycin.

The majority of the tested MRSA isolates (*n* = 24, 70.6%) were multidrug resistant (non-susceptible to at least one agent in three or more of the tested antimicrobial classes, other than β-lactams). In this regard, the non-susceptibility rates, which include both intermediate and resistant isolates, to two, three, four, five, six, seven, and eight antimicrobials were 5.9%, 8.8%, 29.4%, 26.5%, 17.6, 5.9%, and 2.9%, respectively. There was no significant difference in the prevalence of multidrug resistance between the MRSA isolates recovered from environmental surfaces and those isolated from personnel (76.9% versus 66.67%, *P* = 0.704 by Fisher’s exact test).

The full resistance rates were generally higher for personnel isolates than for environmental surface isolates for all tested antimicrobial agents. For cefoxitin, no difference was found between the two specimen categories. For chloramphenicol, doxycycline, erythromycin, and vancomycin, full resistance rates for the environmental surface isolates were higher than their personnel counterparts.

A comparison of the occurrence of antimicrobial resistance among the investigated MRSA isolates in relation to different clonal lineages is presented in Table 7. A significantly higher frequency of ciprofloxacin resistance was recorded among isolates from *spa* type t3689 than other types (χ^2^ = 14.62, df = 7, *P* = 0.0412). Similarly, resistance to clindamycin occurred at significantly higher frequencies in MRSA with *spa* types t3689 or t380 as compared to other types (χ^2^ = 15.8, df = 7, *P* = 0.027). For the remaining antimicrobial agents, non-significant differences in resistance were observed between the *spa* types identified in the current study (Table 7).

**Table 7:** Antimicrobial resistance rates among the investigated MRSA isolates in relation to different clonal lineages.

Antimicrobial agent	No. (%) of MRSA isolates phenotypically resistant to									***P*** **value**
	***spa*** **-CC223**			**Singletons**				**Excluded**	**Total**	
	t223 *n* = 9	t14339 *n* = 8	t3689 *n* = 2	t8506 *n* = 1	t267 *n* = 8	t084 *n* = 3	t1339 *n* = 1	t380 *n* = 2	*n* = 34	
Cefoxitin (FOX)	8 (88.9%)	8 (100%)	2 (100%)	1 (100%)	8 (100%)	3 (100%)	1 (100%)	1 (50%)	32 (94.1%)	0.259
Chloramphenicol (C)	2 (22.2%)	1 (12.5%)	0	0 (0%)	2 (25%)	1 (33.3%)	0	0	6 (17.6%)	0.937
Ciprofloxacin (CIP)	2 (22.2%)	1 (12.5%)	2 (100%)	0 (0%)	0	0	0	0	5 (14.7%)	**0.041**
Clindamycin (CD)	3 (33.3%)	1 (12.5%)	2 (100%)	0 (0%)	0	1 (33.3%)	0	2 (100%)	9 (26.5%)	**0.027**
Doxycycline (DO)	5 (55.6%)	2 (25%)	0	1 (100%)	7 (87.5%)	1 (33.3%)	1 (100%)	2 (100%)	19 (55.9%)	0.081
Erythromycin (E)	6 (66.7%)	5 (62.5%)	0	0	4 (50%)	2 (66.7%)	0	1 (50%)	18 (52.9%)	0.571
Gentamicin (CN)	5 (55.6%)	3 (37.5%)	2 (100%)	1 (100%)	8 (100%)	3 (100%)	1 (100%)	2 (100%)	25 (73.5%)	0.073
Linezolid (LZD)	1 (11.1%)	2 (25%)	2 (100%)	0	1 (12.5%)	2 (66.7%)	0 (0%)	1 (50%)	9 (26.5%)	0.129
Oxacillin (OX)	7 (77.8%)	6 (75%)	2 (100%)	1 (100%)	6 (75%)	0	1 (100%)	1 (50%)	24 (70.6%)	0.209
Vancomycin (VAN)	3 (33.3%)	2 (25%)	0	0	1 (12.5%)	2 (66.7%)	1 (100%)	2 (100%)	11 (32.4%)	0.140

Data indicate the number and percentage (%) of full resistance for each respective antimicrobial agent; isolates showing intermediate resistance to the tested antimicrobials are not included in the table.

All the tested isolates were susceptible to cefaclor, ceftriaxone, imipenem, and neomycin.

*spa-*CC: denotes *spa* clonal complex as determined by the Based Upon Repeat Pattern (BURP) algorithm in the Ridom StaphType software with a distance cost of ≤ 5; excluded: denotes entries excluded from BURP clustering because the *spa* repeat pattern comprised fewer than five repeats.

^*a*^ *P* values calculated using chi-squared tests of 2 × 8 contingency tables. *P* values < 0.05 are highlighted in bold.

#### Prevalence of mecA, mecC, vanA, tst, and PVL-encoding genes

All the tested MRSA isolates possessed the *mecA* gene (*n* = 34, 100%), while *mecC* was not identified.

Ten (29.4%) of the tested MRSA isolates (distributed equally between environmental surfaces and personnel; *n* = 5 for each group) were positive for the *vanA* gene. Four (40%) of these isolates were associated with *spa*-CC223, four (40%) were associated with multiple *spa* types, and two (20%) had a *spa* type (t380) that was excluded from clustering (Tables 2 and 4). The *vanA* gene was detected only in isolates harbouring SCC*mec* type IV (*n* = 7, 70%) and SCC*mec* type I (*n* = 3, 30%) (Table 6).

Seventeen (50%) of the tested MRSA isolates were positive for the *tst* gene (distributed as follows: 9 from environmental surfaces, 6 from hand swabs, and 2 from nasal swabs). These isolates were predominantly associated with *spa*-CC223 corresponding to CC22 (*n* = 12, 70.6%), while the remaining *tst*-positive isolates were associated with *spa* type t267 corresponding to CC80 (*n* = 4, 23.5%) and *spa* type t084 corresponding to CC15 (*n* = 1, 5.9%) (Tables 2 and 4).

Five (14.7%) out of the 34 MRSA isolates contained the *PVL* gene, which seemed to be more associated with environmental surfaces than with personnel isolates (15.38% versus 14.29%), but this difference was non-significant (*P* > 0.9999 by Fisher’s exact test). While all of these *PVL*-positive MRSA isolates carried SCC*mec* type IV (Table 6), the majority (80%, 4/5) were associated with *spa*-CC223, while only one isolate (20%) had a *spa* type (t380) with non-predictable CC (Tables 2 and 4). Additionally, three of the *PVL*-positive strains were also positive for the *tst* gene, one of which also harboured the *vanA* gene (Table 2).

#### Biofilm formation

**Figure 2:**
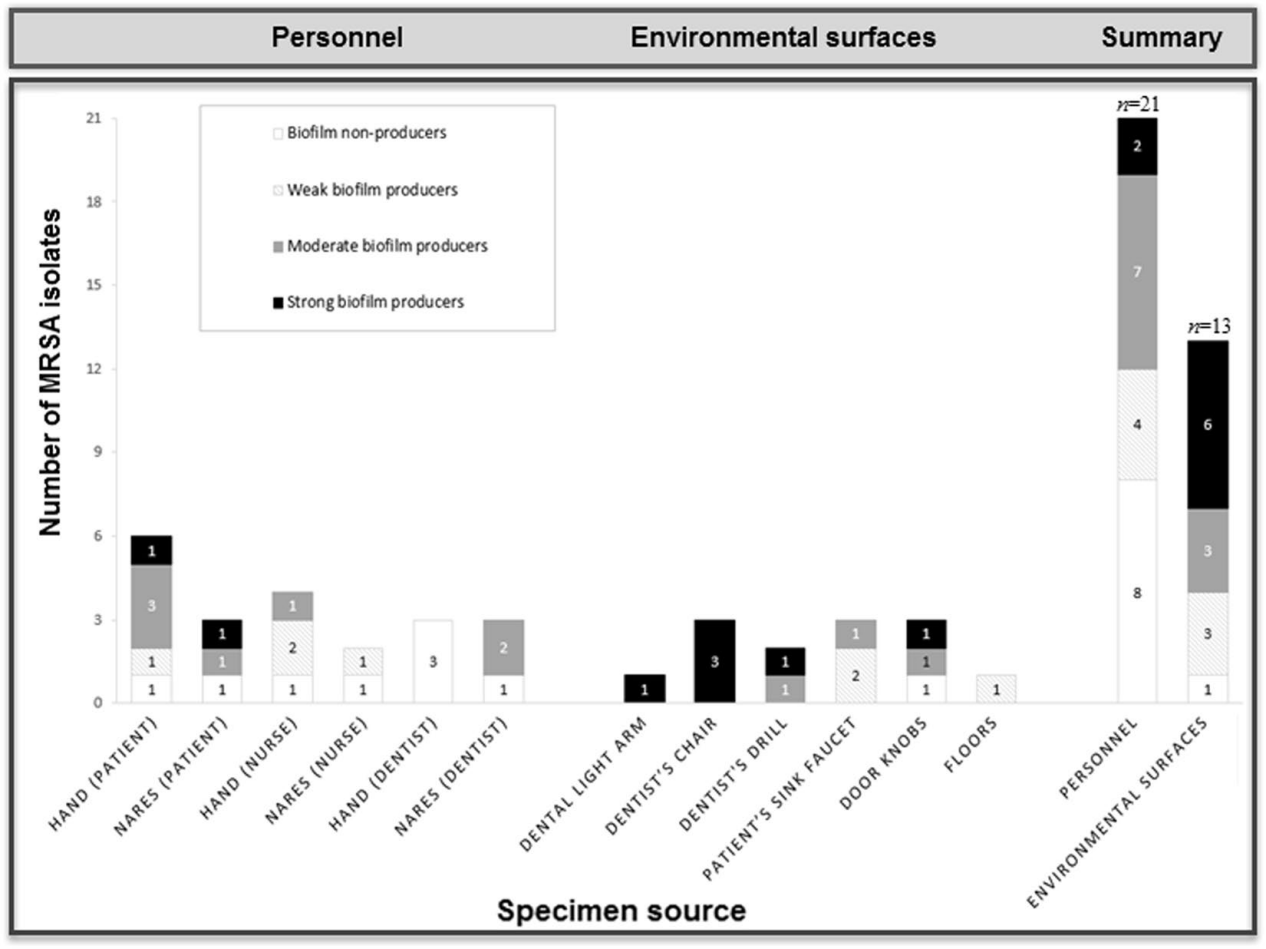
Biofilm-forming abilities of the tested MRSA isolates in relation to the specimen source.

From the 34 MRSA isolates tested for biofilm formation, 8 (23.5%) isolates were classified as strong biofilm producers, 10 (29.4%) were moderate, 7 (20.6%) were weak, and 9 (26.5%) were non-biofilm producers (Fig. 2). This classification was based on the criteria established by Stepanovic and colleagues^34^.

All isolates from personnel were shown to be moderate, weak, or non-biofilm producers, except two isolates with strong biofilm-forming ability (Table 2, IDs: 7 and 93), which were derived from hand and nasal swabs of two different patients attending the prosthetic dentistry and prosthodontic wards, respectively. For isolates recovered from enviromental surfaces, only 7.7% (1/13) of the isolates were non-biofilm formers, while the rest were biofilm formers [46.2% (6/13) strong, 23% (3 /13) moderate, and 23% (3/13) weak] (Fig. 2).

There was no statistically significant difference in biofilm-forming abilities between MRSA isolates recovered from personnel and those recovered from environmental surfaces (χ^2^ = 7.733, df = 3, *P* = 0.0519). Similarly, no significant differences were found in biofilm production between isolates recovered from clinical contact surfaces and those recovered from housekeeping surfaces (χ^2^ = 2.829, df = 3, *P* = 0.4188).

## Discussion

A very limited number of studies, none of which was performed in Egypt, investigated carriage frequency, antibiotic resistance, virulence properties, and genetic diversity of MRSA strains isolated from personnel and environmental surfaces from dental health-care personnel (DHCPs), dental patients, and dental environment. In an effort to fill this knowledge gap, we phenotypically and genotypically characterized MRSA isolates from six different wards at a university outpatient dental clinic in Egypt. We elected to restrict screening of personnel samples to hand and nasal swab specimens, while the environmental surfaces chosen included those commonly encountered within and outside of the patient care area. The recovered isolates were initially identified based on resistance to cefoxitin and oxacillin, and were further confirmed by *mecA* gene detection. Cefoxitin demonstrated a 100% sensitivity for MRSA detection in our hands, as compared to 73.5% in the case of oxacillin, which missed the detection of 10 (26.47%) of the *mecA*-positive isolates (Table 2, IDs: 2, 11, 15, 20, 25, 35, 68, 70, 71, 93). The superiority of cefoxitin for MRSA identification has been reported by a number of authors^35–37^.

In the current study, MRSA represented 3.9% of all recovered bacterial isolates (34/863) and 30.4% (34/112) of all recovered *S. aureus* strains. A 6.6% (12/182) MRSA prevalence was observed among DHCPs, which is in line with the 6.1% prevalence reported among health care workers in the Middle East^38^. This rate is higher than the average global prevalence of MRSA carriage by DHCPs of 4.6%^38^. The MRSA nasal carriage rate among the outpatients screened in the current study (10.2%, 9/88) was lower than the 32% rate reported in a sample of Egyptian outpatients attending primary health care centers [48]. Similarly, the MRSA hand carriage rate among our outpatients (9.8%, 6/61) was much lower than the 47.4% rate obsereved among those attending a dermatology clinic^39^. We detected higher nasal carriage rates of MRSA in outpatients (11.1%, 3/27) than DHCPs (8.2%, 5/61). This is in agreement with the results of a previous study that showed a higher prevalence of nasal MRSA colonization in patients compared with health care workers (5.1% vs. 4.8%)^40^. Overall, our results related to the hand and nasal carriage of MRSA among medical and non-medical personnel, as well as MRSA colonization on surfaces in the clinic environment are consistent with results reported in a number of studies^41–45^. The differences between prevalence rates in our study and others could be attributed to differences in study design, sample size, patient characteristics, and specimen types tested.

Excluding that of prosthodontics, all the investigated wards had one or more of their surfaces positive for MRSA. The absence of MRSA in the tested surfaces of the prosthodontics ward may be attributed to the nature of dental procedures performed in this particular ward, being mostly non-invasive^46^. Supporting this suggestion is the finding that MRSA-positivity of patients’ sink faucets in our study occurred exclusively in the prosthetic dentistry and periodontics wards, where bleeding of patients is common, due to the invasive dental procedures performed in both wards^46^. As might be expected, surfaces with more patient contact (clinical contact surfaces) had higher rates of MRSA colonization than surfaces with less patient contact (housekeeping surfaces). Door knobs were the most contaminated among the investigated housekeeping surfaces. Given the absence of MRSA in housekeeping surfaces that are mainly touched by DHCPs (disinfectant containers, dentist/nurse hand washing sink, and nurses’ desks), it may be reasonable to assume that door knobs in the present study were mainly contaminated by patients’ hand contact. Looking at the possibility of MRSA transmission among the various dental wards and/or among the various specimen categories, only three pairs of isolates showed the same antibiogram (Table 2, IDs: 26 and 44; 63 and 65; and 30 and 95). However, differences were found in the SCC*mec* types and *spa* types carried by the two isolates within each of these pairs.

The majority (*n* = 24; 70.6%) of our MRSA isolates showed multidrug resistance pattern to most of the antimicrobials used. While this may be a reflection of the excessive, unjustified use of broad-spectrum antibiotics in Egypt^47^, this pattern is usually associated with HA-MRSA, because antibiotic resistance in CA-MRSA strains is often limited to β-lactams^48, 49^. Surprisingly, 14 (51.9%) of the multidrug-resistant isolates in our study carried SCC*mec* type IV, which is commonly found in CA-MRSA. The emergence and spread of these multidrug-resistant CA-MRSA isolates could also be the result of the selective pressure of excessive and inappropriate antibiotic usage in our community. In the case of antibiotics known for their potent anti-MRSA activities, the isolates showed a relatively high rate of resistance to vancomycin and linezolid (29.4% and 26.4%, respectively), while there was virtually no resistance to imipenem. Overall, nineteen and thirteen antibiotic resistance profiles were observed among MRSA isolates from personnel and environmental surfaces, respectively. This difference might reflect the presence of strong selective pressure from antibiotic usage in the personnel group.

The investigated isolates were characterized using SCC*mec* and *spa* molecular typing tools. Based on the former typing method, the classical nosocomial SCC*mec* types I and II represented 38.2% and 2.9%, respectively, whereas SCC*mec* type III was completely absent. On the other hand, SCC*mec* type IV (which is usually considered a CA-MRSA marker) was the most predominant type. However, the multidrug resistance profiles and the relatively low prevalence of the *PVL* gene (14.7%) seen in our isolates are also common in HA-MRSA strains. The predominance of SCC*mec* type IV in this study is in agreement with other studies conducted on community-derived and hospital-derived MRSA isolates in the neighbouring territories of Jordan and Gaza^50–52^. Taken collectively, these findings suggest that in Egypt, and probably other neighbouring regions, the population structure of MRSA in the community is starting to mirror that found in the hospital setting, making the boundaries between these two categories so blurred. A similar observation has been made in other countries, which may be attributed to increased MRSA colonization rates in the community, or increased prevalence of nosocomial MRSA^53–59^.

In the current study, *spa*-CC223 was the main *spa* clonal complex (58.8% of the total MRSA isolates). Interestingly, *spa*-CC223 has been reported as the second most predominant *spa*-CC in a study conducted in Kuwait^60^. Additionally, we identified 8 different *spa* types among the tested MRSA isolates, 6 of which (t223, t267, t084, t380, t8506, and t1339) have been previously reported in other Arab countries (Table 4). The limited diversity and the high frequency of the spa type t223 are in agreement with previous data from general population studies as well as from hospital-based studies in the Arab region^50, 61, 62^. This may suggest that certain MRSA clones are more successful than others at surviving, colonizing, and spreading in this geographical region, which is consistent with what has been reported in Europe^63^. The other two remaining *spa* types identified in the current study, namely t14339 and t3689, have been previously reported in isolates from Ireland and Denmark, respectively. Herein, the detection of these two types may be due to the accquision of their respective clones during international travel, or may be a function of the study location in a clinic within a private university, where Arab and non-Arab students from different nationalities are enrolled, some of which have received their secondary education in European countries. Interestingly, the *spa* types from environmental surface isolates were far more clonally conserved than the *spa* types from personnel (4 and 8 different *spa* types, respectively; Table 5). We have also observed differences in the antimicrobial resistance profiles of the strains recovered from the two specimen categories. Taken together, these observations may reflect the different reservoirs of strains to which the two categories are exposed.

Contrary to other studies with similar sample sizes, MRSA isolates in this study showed a limited genetic diversity, with CC80-MRSA-IV-t267 (17.7% of all tested MRSA isolates) and CC22-MRSA-IV-t223 (14.7% of all tested MRSA isolates) being the most predominant clones. While this limited genetic diversity may be attributed to the monocentric nature of the study, the predominance of CC80 and CC22 among the identified clonal complexes is consistent with what has been described previously in studies from Egypt and other neighbouring countries^50, 52, 64, 65^. Additionally, five (14.7%) of the current MRSA isolates (Table 2, IDs: 5, 11, 13, 111, and 112) were identified as *tst*-positive CC22-IV isolates, which are expected to belong to the ‘Middle Eastern variant’ of EMRSA-15. Isolates with similar characteristics have been also reported in studies from Egypt, Italy, the Gaza Strip, Saudi Arabia, Jordan, Kuwait, and the United Arab Emirates^50, 61, 62, 66–69^. Interestingly, the strains reported in Italy and Gaza appeared to be endemic in the tested health care units^66^, and both showed the *spa* type t223, as with the majority of our isolates, suggesting the dissemination of this clone into hospital settings in the Arab and Mediterranean regions. It is noteworthy that the Italian strain exhibited an antimicrobial profile that was different from most of our isolates, because the former was a non-multiresistant MRSA strain. A similar diversity has been shown in a recent study, in which six variants of CC22-MRSA-IV have been detected in the Gulf region^70^. Therefore, the two *tst*-negative CC22-MRSA-IV isolates recovered in the current study (Table 2, IDs: 65, and 103) might be either *tst* deletion mutants of the ‘Middle Eastern variant’ strain or derived from imported European UK-EMRSA-15/Barnim epidemic strain. The latter possibility is supported by the *spa* type (t3689) possessed by of one of the two isolates (Table 2, ID: 65), since it is a common type in Denmark, but not in the Arab region (Table 4). Further investigations are necessary to track the origin of the seven CC22-MRSA-IV isolates described herein, as well as to assess whether they represent a true HA-MRSA clone, such as the classic EMRSA-15, or, alternatively, a CA-MRSA clone that might have spread into the tested dental clinic via the DHCPs or the patients. Future studies should determine risk factors, geographical abundance, transmission patterns, population dynamics, and clinical implications for CC22 strains harbouring the *tst* gene, given the high rate of endemicity characterizing these strains^50, 62, 66, 71^.

An interesting aspect of the current study is the genotypic characterization of the MRSA isolates, which were examined for the presence or absence of five genes (*mecA*, *mecC*, *vanA*, *tst*, and *PVL*) with antimicrobial or virulence-related functions. Given the correlation between antimicrobial resistance and antibiotic consumption^72, 73^, and given the widespread use of non-prescription antimicrobial agents in Egypt^74^, it may not be surprising that *mecA*-positive MRSA strains were recovered from hand and nasal specimens of the outpatients examined in the current study. Additionally, the *mecA*-positive MRSA isolates included strains recovered from DHCPs and environmental surfaces. Both specimen categories are exposed to high antibiotic pressure, caused by being in daily contact with patients receiving antibiotics, with ample opportunity to acquire antibiotic-resistant bacteria, and thus antibiotic resistance genes^43^. Contrary to *mecA*, the *mecC* gene was absent from all isolates. This finding supports previous findings, in which *mecC*-positive MRSA strains have been reported almost exclusively in Europe^75–77^.

When the prevalence of toxin-encoding genes was investigated, five of the tested MRSA isolates were found positive for the *PVL* gene, four of which (80%) were recovered from the hand swabs of DHCPs (including two nurses and two dentists, Table 2). Similarly, seventeen of the tested MRSA isolates were positive for the *tst* gene, five of which (29.4%) were recovered from both nasal and hand swabs of DHCPs (including four nurses and one dentist, Table 2). The hand and nasal colonization of DHCPs with MRSA isolates expressing these toxins is of public health interest, since DHCPs can serve as sources of transmission of these isolates in the community, especially among patients. The clinical implication of this becomes partciulary apparent when considering the carriage of more than one toxin-encoding gene, since three of the investigated isolates carried the gene for *PVL* in combination with the gene for *tst*. One of these three also harboured the *vanA* gene (Table 2, IDs: 11, 111, and 112). While the carriage of both *PVL* and *tst* genes has been reported in a limited number of studies, the majority of these studies^50, 51, 64^, as well as the current one, have been conducted on isolates recovered from the Arab region. This is an alarming observation that needs to be prioritized in the formulation of national and regional health care policies, especially considering the large population exchange between these countries.

The prevalence rate of *PVL*-positive isolates in the present study (14.7%) is comparable to two other studies that have indicated prevalence rates of 15% and 19% among hospital-isolated MRSA strains from Egypt^78, 79^. Similar to previous studies that have shown an association between *PVL*-producing genes and specific MLST CCs^80–82^, the majority (80%, 4/5) of the *PVL*-positive isolates in this study were associated with CC22.

In the case of the *tst* gene, the MLST lineage that showed the strongest association with *tst*-positive strains was also CC22 (70.6%, 12/17) and, to a lesser extent, CC80 (23.5%, 4/17). This fits well with previous studies that have reported the occurrence of *tst*-positive CC22 MRSA strains in Jordan^51, 62^, Kuwait^61^, Saudi Arabia^68^, and the United Arab Emirates^69^, as well as *tst*-positive strains belonging to CC80 in Jordan^51, 64^.

Some MRSA strains are able to produce biofilm on both mucosal and inanimate surfaces, making them difficult to eradicate^83^. Therefore, one of the aims of the present study was to evaluate the biofilm-forming ability of the tested isolates. The results showed that among the eight MRSA isolates recovered from nasal swabs, one (12.5%) isolate was classified as strong biofilm producer, three (37.5%) were moderate, one (12.5%) was weak, and three (37.5%) were non-biofilm producers. This is consistent with the general idea that a dispersed mode of growth is favoured over a biofilm mode during *S. aureus* nasal colonization^84^. Interestingly, three of the five isolates that are likely to be related to the ‘Middle Eastern variant’ of EMRSA-15 were strong biofilm producers (Table 2, IDs: 5, 11, and 13). Collectively, the detection of multiple virulence and antimicrobial resistance genes suggests the pathogenic potential of the MRSA isolates in the current study, especially when combined with their ability to form biofilms, and thus their potential to resist disinfectants or sanitizers.

This is the first study to provide an overview of MRSA clones currently circulating among patients, DHCPs, and environmental surfaces in dental clinics in Egypt. The main findings of this study include: (i) the limited genetic diversity of MRSA isolates within dental clinics in Egypt (ii) the detection of five *tst*-positive and two *tst*-negative CC22-IV isolates that are likely to be linked to the epidemic EMRSA-15 clone; (iii) the combined occurrence of *tst* and *PVL* in three of the isolates; (iv) the high level of multidrug resistance in the isolates; (v) the predominance of SCC*mec* type IV-harbouring MRSA isolates in the population; (vi) the blurring of traditional distinctions between CA-MRSA and HA-MRSA based on SCC*mec* type and antibiotic resistance in the community, which may suggest the infiltration of CA-MRSA into the hospitals in the area; and (vii) the detection of isolates with *spa* types (t14339 and t3689) that have never been reported before in any Arab country. In conclusion, the results suggest that personnel and dental clinic surfaces may serve as sources for transmission of MRSA. They can also act as important reservoirs for antibiotic resistance genes. Results reinforce the need for continuous national and regional MRSA surveillance programmes in order to keep track of the emerging clones. Strict antibiotic policy and infection control measures should be implemented to reduce the incidence of MRSA infection in dental clinics and other health care settings.

## Materials and Methods

### Study design and sample collection

In the present cross-sectional, monocentric study, a total of 1300 samples were collected from six different dental wards of a university outpatient dental clinic in Egypt, between January and May 2013. Samples collected were obtained from: (i) the hands and anterior nares of patients and DHCPs; and (ii) environmental surfaces within the clinic. The six dental wards included the endodontics, operative dentistry, periodontics, prosthetic dentistry, prosthodontics, and dental surgery wards^19^.

All samples collected in this study (whether from personnel or environmental surfaces) were obtained during the working hours of the clinic. Participants were chosen randomly to differentiate CA-infections from HA-infections. A written informed consent was obtained from each subject. The study protocol was approved from the Ethics Committee of the Faculty of Pharmacy, October University for Modern Sciences and Arts (MSA). All methods were performed in accordance with the relevant guidelines and regulations. Hand swabs were collected during working days, immediately after removing the gloves (if applicable) and before washing. For sampling, the palms and periungual areas were vigorously rubbed with sterile saline-moistened cotton swabs. Paired nasal swabs were collected from each participant according to a previously described method^85^.

The environmental surfaces investigated in this study were categorized into two groups: (i) the clinical-contact surfaces (that is, surfaces that are touched frequently during dental procedures), which included: dental light arms, dentists’ chairs, dentists’ drills, dentists’ tool racks, patients’ sink faucets, and X-ray switches; and (ii) the housekeeping surfaces (surfaces outside of the patient care area), which included: dentist/nurse hand-washing sinks, disinfectant containers, door knobs, floors away from the dentists’ chairs, light switches, and nurses’ desks.

One set of environmental surface samples from each of the investigated wards included all the above-mentioned areas from both clinical contact and housekeeping surfaces. These samples were collected at the end of the clinic hours (before general cleaning for the next day) and following the CDC guidelines for environmental infection control^86^. Briefly, each sample was collected by applying a sterile water-moistened swab firmly over an approximate area of 5 × 20 cm of the specific object. In wards with two to twelve chairs, two chairs were randomly chosen, and samples were taken from both chairs, while in wards with two dental chairs, samples were collected from both chairs.

### Identification of *S. aureus* and screening for methicillin resistance

Swabs (collected from hands, anterior nares, or environmental surfaces) were inoculated into 2 ml of double strength brain heart infusion broth (BHI; Difco, USA), and incubated at 37 °C for 18–24 h. A volume of 100 μl was withdrawn from cultures showing growth, plated onto mannitol salt agar (Difco, USA), and the plates were incubated aerobically at 37 °C for 24 h. The yellow-colored colonies on mannitol salt agar were collected for further identification using standard microbiological methods. These methods included colony morphology on blood agar, Gram stain, in addition to catalase and coagulase tests. Strains with ambiguous biochemical results were analyzed by 16 S rRNA gene sequencing as described elsewhere^87^.

All *S. aureus* isolates were screened for methicillin resistance using oxacillin (1 µg) and cefoxitin (30 µg) disc diffusion tests. Briefly, bacterial cultures were adjusted to the 0.5 McFarland turbidity standard, which is equivalent to 1.5 × 10^8^ CFU/ml, and inoculated (using a sterile cotton swab) on the surface of a Mueller Hinton Agar (MHA) (Oxoid, UK) in the case of cefoxitin, or MHA supplemented with 2% sodium chloride in the case of oxacillin. Zone diameters were measured and interpreted according to the guidelines of the Clinical Laboratory Standard Institute (CLSI)^88^. All MRSA isolates were stored at −20 °C in BHI containing 15% glycerol for further characterization.

### Molecular typing methods

#### SCCmec typing

A multiplex PCR with five primer-pairs (Table 8) was used as previously described^89^ to discriminate between SCC*mec* types I, II, III, IV, and V. The SCC*mec* type was determined on the basis of the band pattern obtained. Isolates with no visible bands, or with a band pattern that was not in agreement with one of the five predicted band patterns, were classified as non-typeable.

**Table 8:** PCR primers used in this study.

Target gene (Primer specificity)	Primer sequence (5’ → 3’ direction)^*^	Amplicon size (bp)	Annealing temperature (^°^C)	Reference
*mecA*	Forward: GAAGATGGCTATCGTGTCACA Reverse: GGAACTTGTTGAGCAGAGGTT	307	52	Current study
*mecC*	Forward: GGGTTCAGCCAGATTCATTTGT Reverse: GTACTGTTGCTTCGTTCAATGG	138	52	Current study
*spa*	1113_ Forward: TAAAGACGATCCTTCGGTGAGC 1514_ Reverse: CAGCAGTAGTGCCGTTTGCTT	Variable (180–670)	52	91
*PVL*	Forward: GCTGGACAAAACTTCTTGGAATAT Reverse: GATAGGACACCAATAAATTCTGGATTG	83	50	94
*VanA*	Forward: GGGAAAACGACAATTGC Reverse: GTACAATGCGGCCGTTA	732	50	95
SCC*mec* I	CIF2 F2: TTCGAGTTGCTGATGAAGAAGG CIF2 R2: ATTTACCACAAGGACTACCAGC	495	47	96
SCC*mec* II	KDP F1: AATCATCTGCCATTGGTGATGC KDP R1: CGAATGAAGTGAAAGAAAGTGG	284	47	96
SCC*mec* III	RIF5 F10: TTCTTAAGTACACGCTGAATCG RIF5 R13: GTCACAGTAATTCCATCAATGC	414	47	96
SCC*mec* I, II, IV	DCS F2: CATCCTATGATAGCTTGGTC DCS R1: CTAAATCATAGCCATGACCG	342	47	96
SCC*mec* V	ccrC F2: GTACTCGTTACAATGTTTGG ccrC R2: ATAATGGCTTCATGCTTACC	449	47	89
*tst*	Forward: ACCCCTGTTCCCTTATCATC Reverse: TTTTCAGTATTTGTAACGCC	326	47	97

^*^F: forward primer; R: reverse primer.

#### spa typing and Based Upon Repeat Patterns (BURP) analysis

Using the primers listed in Table 8, all the investigated MRSA isolates (*n* = 34) were subjected to a PCR assay for amplification of the polymorphic repeat region (X region) of the *spa* gene as described elsewhere^90^. The *spa* amplicons were then purified using a Qiagen DNA purification kit (Qiagen GmbH, Hilden, Germany) and sequenced by Macrogen® (Seoul, South Korea) using capillary electrophoresis. *spa* typing was conducted as described by Harmsen *et al*.^91^, and the resulting *spa* types were then clustered into related *spa* clonal complexes (*spa*-CCs) using the BURP algorithm implemented in the Ridom StaphType software version 2.2.1 (Ridom GmbH, Würzburg, Germany). The default parameters of the BURP algorithm (exclusion of *spa* types shorter than 5 repeats and clustering of *spa* types if cost is less or equal to 5) were applied in this analysis, in order to prevent the formation of *spa* clusters that are too large or non-specific^92^. The *spa* type that could not be assigned to a cluster was considered as a singleton. Due to the high concordance between *spa* typing and MLST^24^, the MLST clonal complexes (CC) corresponding to the respective *spa*-CCs were deduced from the data on the Ridom SpaServer (http://spaserver.ridom.de, last accessed on 15 November 2016) and by literature search.

#### Antimicrobial susceptibility testing

Excluding vancomycin and linezolid, the antimicrobial susceptibility of the isolates to a panel of commonly used antibiotics was determined using the Kirby Bauer disc diffusion method on MHA plates according to CLSI guidelines and breakpoints^88^. The antimicrobial discs used, which were all obtained from Oxoid (UK), included: cefaclor (CE; 30 µg), cefoxitin (FOX; 30 μg), ceftriaxone (CRO; 30 μg), chloramphenicol (C; 30 μg), ciprofloxacin (CIP; 5 μg), clindamycin (CD; 2 µg), doxycycline (DO; 30 µg), erythromycin (E; 15 μg), gentamicin (CN; 10 μg), imipenem (IMP; 10 µg), neomycin (NE; 30 µg), and oxacillin (OX; 1 μg). The susceptibilities of the isolates to vancomycin and linezolid (both from Sigma Aldrich, Germany) were determined using the agar dilution method following the CLSI guidelines and interpretative criteria^88^. Throughout the study, the antimicrobial susceptibility tests were quality controlled using *S. aureus* ATCC 43300 (methicillin-resistant strain) and *S. aureus* ATCC 29213 (methicillin-sensitive strain).

#### Detection of mecA, mecC, vanA, tst, and PVL-encoding genes

Presumptive MRSA isolates were confirmed by polymerase chain reaction (PCR) using primers targeting the *mecA* and the *mecC* genes. Additionally, all the MRSA isolates were subjected to a PCR assay for detecting the *lukF/S-PV* genes encoding the PVL toxin and the *tst* gene encoding the TSST-1 toxin, while only those found to be phenotypically resistant or intermediately resistant to vancomycin were tested for the presence of *vanA* gene. The primers used in these assays (Table 8) were synthesized by Eurofins MWG Operon (Ebersberg, Germany). Genomic DNA extraction and purification was done using the GeneJET^®^ Genomic DNA purification kit (Thermo Scientific, USA) according to the instructions of the manufacturer. Each PCR amplification cycle consisted of an initial denaturation step at 95 °C for 10 minutes, followed by denaturation at 95 °C for 30 s, annealing at 47–52 °C (depending on primers used, Table 8) for 30 s, and extension at 72 °C for 1 min for each kb of DNA amplified. This cycle was repeated 35 times followed by a final extension step at 72 °C for 10 minutes. The final volume of the reaction mixture for each PCR assay was 25 µl, and amplifications were performed using the Biometra TAdvanced thermal cycler (Biometra, Göttingen, Germany). All PCR-based assays employed known positive and negative controls. After amplification, 10 µl of each PCR reaction was separated on a 2% (w/v) agarose gel, stained with ethidium bromide (0.5 mg/ml), and visualized under a Gel Doc EZ Imager (Bio-Rad Laboratories, USA).

#### Biofilm formation assay

The ability of the MRSA isolates to form biofilm onto polystyrene microtiter plates was evaluated as described previously with slight modifications^93^. Briefly, overnight bacterial cultures in trypticase soy broth (TSB, Difco, USA) were diluted in the same medium to match the 0.5 McFarland turbidity standard, followed by further dilution (1:100) in TSB supplemented with 2% (w/v) glucose and 2% (w/v) NaCl. A volume of 200 µl of this diluted bacterial suspension was cultured in triplicates in microtiter wells (96 wells; Nunc, Denmark), while negative control wells contained uninoculated medium. The plates were incubated at 37 °C for 24 h. Following incubation, the plates were washed carefully three times with 200 μl of tryptone water (Difco, USA) to remove nonadherent planktonic cells, and the plates were subsequently dried at room temperature. The established biofilm was stained with 100 μl/well of 0.1% membrane filtered crystal violet solution (Sigma Aldrich, Germany) at room temperature for 2 min. The wells of the microtiter plates were then washed twice with sterile pyrogen-free water, and finally a mixture of ethanol:acetone (4:1, v/v) was used to elute bound crystal violet. The eluted crystal violet was diluted 1:10 with the same mixture of solvents, and the optical density was determined spectrophotometrically at λ = 545 nm using microplate ELISA reader (Stat Fax^®^2100). The isolates were classified as biofilm non-producers, weak, moderate, and strong biofilm producers based on previously published criteria^34^.

### Statistical analysis

Categorical variables were compared using the Chi-square test (χ2) or Fisher’s exact two-tailed test, as appropriate, with *P* values of < 0.05 as the level of significance. These statistical analyses were carried out using the GraphPad Prism (version 6; GraphPad Software Inc.; USA).
